# Various nanoparticle morphologies and surface properties of waterborne polyurethane controlled by water

**DOI:** 10.1038/srep34574

**Published:** 2016-09-30

**Authors:** Xing Zhou, Changqing Fang, Wanqing Lei, Jie Du, Tingyi Huang, Yan Li, Youliang Cheng

**Affiliations:** 1 School of Mechanical and Precision Instrument Engineering, Xi’an University of Technology, Xi’an 710048, P. R. China; 2 Faculty of Printing, Packaging Engineering and Digital Media Technology, Xi’an University of Technology, Xi’an 710048, P. R. China; 3 College of Art and Design, Xi’an University of Technology, Xi’an 710048, P. R. China; 4 School of Civil Engineering and Architecture, Xi’an University of Technology, Xi’an 710048, P. R. China

## Abstract

Water plays important roles in organic reactions such as polyurethane synthesis, and the aqueous solution environment affects polymer morphology and other properties. This paper focuses on the morphology and surface properties of waterborne polyurethane resulting from the organic reaction in water involving different forms (solid and liquid), temperatures and aqueous solutions. We provide evidence from TEM observations that the appearance of polyurethane nanoparticles in aqueous solutions presents diverse forms, including imperfect spheres, perfect spheres, perfect and homogenous spheres and tubes. Based on the results on FTIR, GPC, AFM and XRD experiments, we suggest that the shape of the nanoparticles may be decided by the crimp degree (i.e., the degree of polyurethane chains intertangling in the water environment) and order degree, which are determined by the molecular weight (*M*_*n*_) and hydrogen bonds. Meanwhile, solid water and high-temperature water can both reduce hard segments that gather on the polyurethane film surface to reduce hydrophilic groups and produce a soft surface. Our findings show that water may play key roles in aqueous polymer formation and bring order to molecular chains.

Waterborne polyurethane (WPU) dispersions play a particularly important role in a wide range of industrial coatings and adhesive applications due to their versatile performance generated by variations of soft and hard segments. It is the only class of polymers that displays thermoplastic, elastomeric and thermoset behaviour^1^. In pursuit of WPU dispersions with better properties and appearance, many publications^2^,^3^,^4^,^5^,^6^ have provided instructions for preparing WPU products, e.g., describing the impact of the type and content of ion, catalyst, isocyanate, oligomer polyol, chain extender and neutralization reagent, among other factors, on the properties of the dispersions. However, water, one of the most important components in WPU synthesis, has been largely ignored due to its monotonous category and apparently simple structure. In fact, whether a WPU dispersion remains homogenous or becomes high-performance in aqueous environments should intrinsically rely on the interaction of the WPU prepolymer with water. Thus, the main focus of this work was to reveal the impact of water on the morphology of WPU nanoparticles and surface properties of WPU films.

Recently, the formation of nanosized latex particles with special morphology has become increasingly attractive in both academic and industrial research. It is believed that small latex particles, which can improve the appearance, mechanical properties^7^ and quick-drying property via deep penetration of the dispersion into a substrate^8^, have a wide range of industrial applications, such as film-forming materials, additives for inks or water-based inks^9^,^10^. According to Runt *et al.*^11^, the morphology of polyurethane consists of both randomly oriented cylinders and domains with the general appearance of “spheres”, which are most likely a combination of spherical domains and portions of cylindrical domains. Kim and Park *et al.*^12^ reported that a type of rod-coil molecule consisting of hydrophilic and hydrophobic parts can self-assemble into a variety of architectures in aqueous solution, such as spherical particles, capsules, fibres, tubes and ribbons. As expected, the hard/soft segments are the hydrophilic/hydrophobic parts in WPU chains, respectively. Meanwhile, Li and Sun^13^ reported that only polyurethane dispersions synthesized with oligomer polyols of molecular weights of 1000 and 2000 g/mol resulted in spherical particles when dispersed in water. Notably, when preparing latex particles, water plays a key role in determining the formation and morphology of such nanosized particles. Water is an interesting molecule in chemistry due to its fascinating array of unusual properties in pure form and as a solvent^14^. Therefore, a large amount of research has been focused on the study of water^15^,^16^,^17^ and its function in reactions, especially in protein aggregation^18^,^19^ and organic reactions^20^. Bulk water exists in many forms, such as liquid, vapour and numerous crystalline and amorphous phases of ice, with hexagonal ice being responsible for the fascinating variety of snowflakes^21^. Due to the absorbance at interfaces and confinement in microscopic pores of water molecules, water can determine various properties in materials science, geology, biology, tribology and nanotechnology^21^,^22^,^23^,^24^. All forms of water, including liquid, vapour, ice and snowflakes, may be used as reagents to obtain unexpected properties. Although some studies have been devoted to WPU synthesis and nanoparticle morphology^7^,^8^,^9^,^10^,^11^,^12^,^13^,^16^, the influence of water as a chain extender, emulsifier and solvent on the shape of WPU particles and film surface remains unexplored. In this study, water was examined in different forms and at different temperatures to study this influence. Here, we report the morphology detected by transmission electron microscopy (TEM) and atomic force microscopy (AFM) and the surface properties of WPU films resulting from the reaction of polyurethane prepolymer in water in the forms of liquid (at different temperatures), ice and snowflakes. In an aqueous environment reaction, we show that water–the oft ignored component of waterborne polyurethane–also plays a structural role by adjusting the molecular chains and strength of hydrogen bonds in the polyurethane nanoparticles.

## Experiment

### Materials

Isophorone diisocyanate (IPDI, 98 wt% purity, purchased from Jingchun Chemical, Shanghai, China), which is liquid at room temperature, was used. Poly (neopentyl glycol adipate) (PNA, molecular weight (M_w_) = ca. 2000) was used as the oligomer glycol to synthesize polyurethane samples (dried under vacuum at 120 °C before use). Dimethylolpropionic acid (DMPA, purchased from Jingchun Chemical, Shanghai, China) was employed as a hydrophilic chain extender. 1,4-butanediol (BDO, 99.5 wt% purity), potassium hydroxide (KOH, 85 wt% purity) and triethylamine (TEA, 99 wt% purity) were purchased from Fuchen Chemical (Tianjin, China). Dibutyltin dilaurate (DBTDL) was purchased from Qingxi Chemical (Shanghai, China). Small doses of acetone was used throughout the traditional process. Deionized water was used at different temperatures and in different forms.

### Synthesis of waterborne polyurethane by deionized water at different temperatures and in different forms

Waterborne polyurethane (WPU) dispersion samples were synthesized by the addition of deionized water or hydrogen peroxide with a hard-/soft-segment molar ratio of 4 and the appropriate NCO/OH ratio of 1.2^9^. The DMPA content was set to 5 wt% (with respect to the prepolymer weight)^25^. PNA and IPDI were added to a four-necked flask (500 mL) equipped with a mechanical stirrer, thermometer and spiral condenser in an electric-heated thermostatic water bath. The reaction was performed at 85 °C for 3 h at a stirring rate of 200 rpm. DBTDL was added when the first reaction had occurred for 2 h, followed by the addition of DMPA at 60 °C. The reaction was continued at 85 °C for another 2 h at a stirring rate of 400 rpm. The resulting prepolymer was then cooled to approximately 20 °C; BDO was then added into the flask with a small amount of acetone, and the reaction continued for 1 h. Next, to prepare different WPU samples, the same weight of deionized water at different temperatures and in different forms was poured into the flask at a stirring rate of 1000 rpm for emulsification, chain extension and dispersion of the polyurethane prepolymer. In preparing WPU, a small amount of acetone (acetone and water at a ratio of 1:80 or less with respect to the stoichiometric relationship in volume) was added to reduce the viscosity of the prepolymer. KOH was used as a neutralizer to adjust the pH of both samples to 8.5–9.0. After the reaction, the residual acetone in the WPU samples was removed in a vacuum drying oven at 50 °C and 0.05 MPa for 1 h. The films were obtained by casting the WPU samples onto Teflon surfaces, followed by a slow evaporation of the solvent at room temperature for 3 days and then at 40 °C in a vacuum drying oven for 12 h to completely remove the solvent. Then, the Teflon films were stored in a desiccator to avoid moisture. In this experiment, a suitable relative humidity (RH) of approximately 70% was ensured. The abbreviations of the WPU samples synthesized from snow, ice, a mixture of ice and deionized water and deionized water at different temperatures (20 °C, 50 °C, 80 °C) are samples S-WPU, I-WPU, IW-WPU and W-WPU (1, 2, 3), respectively. Since water at ca. 20 °C (common room temperature) is normally used in waterborne polyurethane synthesis, the WPU sample (W-WPU1) obtained from 20 °C water was used as the point of comparison to represent the normal and general bulk structure and nanoparticle morphology^26^,^27^.

### Characterization and property measurements

Fourier transform infrared spectroscopy (FTIR) was used to identify the structure of the WPU. The infrared spectra of the dried polyurethane films were obtained using a Fourier transform IR spectrophotometer (SHIMADIU FTIR-8400S (CE)) and recorded in transmission mode at room temperature by averaging 20 scans at a resolution of 16.0 cm^−1^. The spectra were analysed in the frequency range of 4000-400 cm^−1^. An X-ray diffractometer (XRD) instrument (XRD-7000, SHIMADZU LIMITED, Japan) was used to analyse the crystallinity of the polyurethane films with monochromatic Cu K_*α*_radiation (1.540598 nm). The 2θ angles between 10° and 60° were scanned at a rate of 8.0000 deg/min. The polymer molecular weights (number-average molecular weights (*M*_n_) and weight-average molecular weights (*M*_w_)) and molecular weight distributions were determined by gel permeation chromatography (GPC) (USA Waters, ALLIANCE) analysis with a DAWN EOS (λ = 690.0 nm) and RI detector (Shodex RI-71). Molecular weights were relative to monodisperse polystyrene standards, and terahydrofuran (THF) was used as the eluent with a flow rate of 0.500 mL/min. Transmission electron microscopy (TEM) was performed to investigate the microstructures of the WPU samples using a JEM-3010 microscope with a Gatan894 CCD camera operating at an accelerating voltage of 300 kV. The samples were prepared by diluting one drop of dispersion in deionized water to a concentration of 1 wt%. The water contact angle (WCA) was measured on an OCA 20 (Dataphysics, Germany) with a water drop volume of 2 μL. Atomic force microscopy (AFM) was performed to determine the nanostructural and nanomechanical properties of the WPU films using atomic force microscopy (Dimension Icon, BRUKER, Germany) in the imaging and tapping modes, respectively. All the AFM experiments were performed in air at room temperature, and the height and phase images (scan sizes ranging from 10 μm × 10 μm to 5 μm × 5 μm and 256 pixels × 256 pixels) were acquired using a scan rate of 1.56 Hz. To clearly investigate the interaction between the hard and soft domains in WPU, the nanomechanical properties of the WPU samples were examined by AFM force spectroscopy (FS) experiments using contact mode at room temperature. To prepare the WPU films, approximately 5 mm × 5 mm pieces were cut from the cast films on Teflon sheets and mounted onto the AFM sample holder. A grid of 20 points × 20 points on a 2 μm × 2 μm area of the WPU films was chosen to give a final map of 400 points^28^. In each FS experiment on the WPU films, FS curves were recorded at each point on the grid for a total of 400 points to produce a data set with statistical significance. The other parameters of the AFM experiment were set as before^28^,^29^. Due to the detection mode set, it was necessary to transform the FS curves from force-scanner (*F-Z*) curves to force-distance (*F-d*) curves, which could more directly reflect the nanomechanical properties of the WPU films. The true distance (*d*) between the surface and the AFM tip was calculated by subtracting the deflection of the cantilever (*Z*) from the height values corresponding to the measured piezoelectric displacement, *Z*_piezo_:




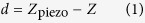




### Nanomechanical properties

Multiple nanomechanical properties, such as adhesion, dissipation, Young’s modulus and deformation of the WPU films, can be analysed and compared qualitatively using the *F-d* curves. The adhesion of WPU films is regarded as the maximum adhesive force of the film surface to the tip, which is the force value between the retraction line and base line, abbreviated *F*_max_. *F*_max_ is primarily a measure of the force required to deform polyurethane in the vicinity of the tip’s contact area and thus could be applied to compare the adhesive strength among the WPU samples qualitatively but not quantitatively. The adhesion dissipation of WPU films is related to the viscoelasticity; this is reflected by the non-elastic deformation of the AFM cantilever beam, *W*, which was calculated by the following equation:




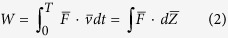




where *W* is the area between the axis *F* = 0 and the retraction force-displacement curve, 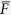 is the force on the AFM cantilever beam, *T* is the recording time and 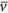 and 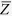 are the moving speed and moving distance of the AFM cantilever beam, respectively. The deformation property of the WPU films was defined as the maximum distance measured from the initial surface position, abbreviated *d*_max_. According to the previously proposed elastic contact models^29^,^30^,^31^, the Young’s modulus could be obtained using analytical expressions. First, the spring constant *k* was obtained. According to the BRUKER force curve instructions, the Sader method is appropriate to calculate the value, as follows:




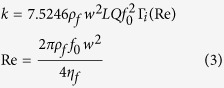




where *L* and *w* are the length and width of the cantilevers, respectively, which can be measured by the standard grating from the microscope; *f*_*0*_ is the resonance frequency; and *Q* is the quality factor. The latter values could be acquired using the Thermal Tune method in BRUKER software. During the detection process, we controlled for the elastic response by observing the indentation area after measuring the force. Meanwhile, to ensure an absence of indentation marks, normal loads had to be selected. Next, all the Young’s moduli of the WPU were calculated to evaluate the influence of water in different forms and at different temperatures on the nanomechanical properties.

## Results and Discussion

**Figure 1:**
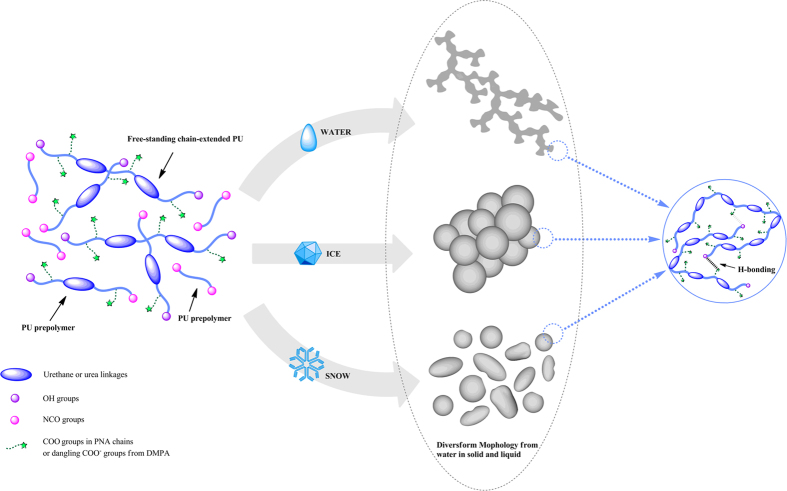


Anionic waterborne polyurethane dispersions were synthesized in a prepolymer process using ca. 2000 g/mol PNA (PNA-2000) as the soft segment and IPDI as the diisocyanate. Water in different forms (snow, ice, mixture of ice and water) and at different temperatures (20 °C, 50 °C, 80 °C) was employed to study the morphology of the polyurethane nanoparticles. The synthesis route of the WPU samples is shown in Fig. 1. All the samples had an equal concentration of hard and soft segments based on the molar ratio of 4 based on previous research by our group^6^,^9^.

**Figure 2:**
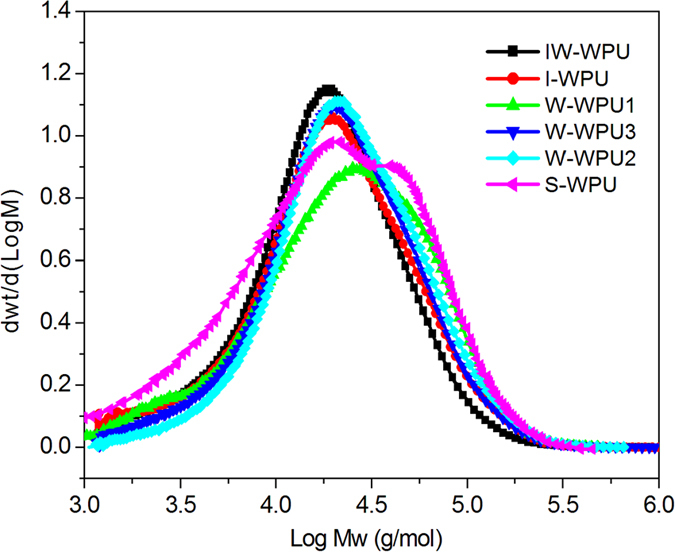


The molecular weights and molecular weight distributions were determined by GPC using THF as the mobile phase and polystyrene as a reference, as shown in Fig. 2. The average molecular mass (*M*_*n*_), the weight average molecular mass (*M*_*w*_) and the polydispersity index (PDI) of the polyurethanes obtained by GPC are also summarized in Table 1. The *M*_*n*_ sequence of the polyurethanes was W-WPU2 > S-WPU > W-WPU1 > W-WPU3 > IW-WPU > I-WPU, while the *M*_*n*_ sequence of the PDI was I-WPU > W-WPU1 > S-WPU > IW-WPU > W-WPU3 > W-WPU2. The resulting PDI data indicated that the molecular weight distribution of the polyurethane dispersion obtained from ice was the broadest, and that obtained from 50 °C deionized water was the narrowest. Meanwhile, the molecular weight of W-WPU2 was the largest, and the molecular weight of I-WPU was the smallest, suggesting that the polyurethane synthesized from 50 °C deionized water has the longest molecular chain. The molecular weight and molecular weight distribution results indicate that deionized water at 50 °C is ideal for the chain extension, emulsification and dispersion of the polyurethane prepolymer when a large molecular weight and narrow molecular weight distribution are desired. It was confirmed that the molecular weight was significantly influenced by the isocyanate-water reaction, which is favoured at the oil/water interface. The molecular weight increases when the extent of the reaction between isocyanate and water decrease^32^. Thus, the 50 °C deionized water molecule at the oil/water interface might reduce the isocyanate-water reactions to generate long and homogenous polyurethane molecule chains compared with the polyurethane dispersions obtained using deionized water in other forms and at other temperatures. The WPU samples synthesized with ice had relatively low molecular weights, possibly due to the increase in the isocyanate-water reaction that arises from the slow melting of ice into water. As depicted in Fig. 2, no significant differences were observed between the molecular weight distributions of all samples, except for S-WPU. The S-WPU distribution function was asymmetric and presented a bimodal distribution, which can be attributed to the formation of allophanate units or chain propagation^33^. Furthermore, there was a relationship between the isocyanate-water reaction (molecular weight and molecular weight distribution) and the particle size of polyurethane; for example, upon increasing the particle size, the isocyanate-water reaction, which is favoured at the oil/water interface, was reduced compared to the isocyanate-hydroxyl reaction, which is favoured in the particle core^32^,^34^. These results suggest that the molecular weight of polyurethane, but not the molecular weight distribution, affects the particle size of polyurethane dispersions. This effect will be analysed in the TEM experiment section.

**Figure 3:**
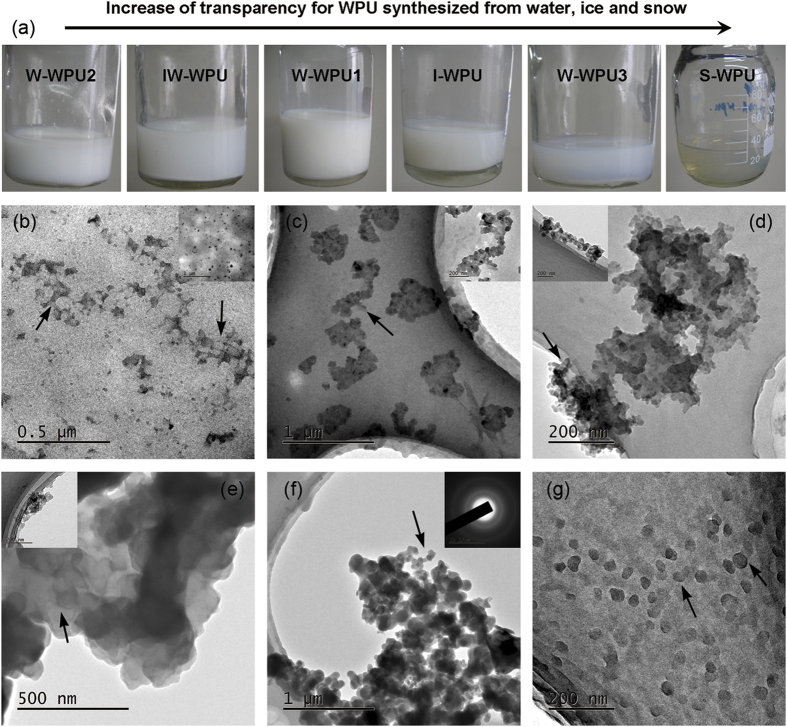
Digital photos and morphology of waterborne polyurethane dispersions prepared by water can be diversiform and is mainly spherical from TEM experiments. (**a**) Digital photos waterborne polyurethane dispersions; (**b**–**d**) (W-WPU1, 2, 3), Observations of synthetic polyurethane nanoparticles by deionized water in different temperature: (**b**) Dispersion obtained with 20 °C deionzied water presents unregular nanoparticles and the micro formation of film. (**c**) Dispersion obtained with 50 °C deionzied water show regular nanoparticles in rectangle. (**d**) Dispersion obtained with 80 °C deionzied water presents spherical nanoparticles from ca. 10 nm–100 nm. (**e–g**) (IW-WPU, I-WPU, S-WPU), Observations of polyurethane dispersion particles by crystalline water: (**e**) Dispersion obtained with mixture of ice and water show imperfect spherical nanoparticles. (**f**) Dispersion obtained with ice present perfect spherical nanoparticles. (**g**) Dispersion obtained with snow show homogeneous and perfect spherical nanoparticles.

**Figure 4:**
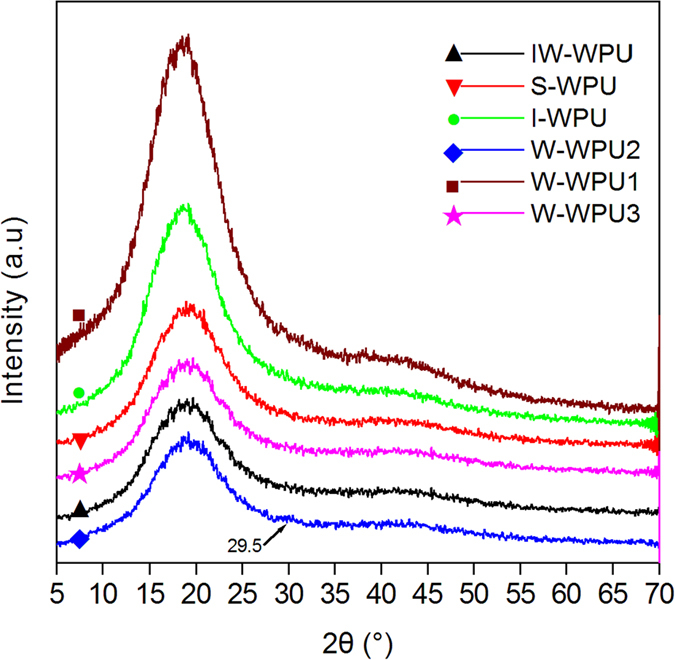


To observe the morphology of these WPU nanoparticle dispersions, a TEM experiment was performed using the films on the grids. As shown in Fig. 3(e–g), the particles in the three samples presented spherical or related shapes, some of which are depicted in Fig. 1. WPU1 and WPU3 tended to form spherical particles, while WPU2 particles showed rectangular aggregation. The size of the nanoparticles ranged from 10 nm to 286.7 nm (Fig. 3(b,d–g)). Furthermore, as shown in Fig. 3, some of the spherical particles aggregated together and formed blocks of membrane. The aggregation of nanoparticles shown in Fig. 3 (cartoon in Fig. 1) is essentially consistent with the kinetic modelling of aggregation and gel formation in quiescent dispersions of polymer colloids postulated by Morbidelli *et al.* and confirmed by Madbouly *et al.*^26^,^34^. In terms of this model, the aggregates interconnect directly with their nearest neighbours to form a network occupying the total volume of the dispersion. This aggregation, depicted in Fig. 3(b,d,e), notably reflects the formation process of polyurethane films. As a comparison, the appearance of the W-WPU1 nanoparticles shown in Fig. 3(b) is irregular, a type of imperfect sphere. The nanoparticles of W-WPU3, IW-WPU, I-WPU and S-WPU depicted in Fig. 3(d–g) appear as spheres or even perfectly homogenous spheres. In addition, there are some irregular and fuzzy domains adjacent to the perfectly homogenous sphere in Fig. 3(g) that could be ascribed to the allophanate units or chain propagation described in the GPC results. The most different nanoparticle was W-WPU2, as depicted in Fig. 3(c), which shows regular blocky structures that aggregate together to form bulky irregular shapes. The WPU nanoparticle morphologies in Fig. 3 clearly show that the dispersions obtained using solid water (snow and ice) present spherical nanoparticles in a higher degree than liquid water. Therefore, we suggest that solid water might induce polyurethane chains to form spherical particles. This behaviour may result from the reactivity of water at different temperatures and in different forms. The sequence of the samples according to the degree of regularity of the spheres shown in Fig. 3 was S-WPU > I-WPU > IW-WPU ≥ W-WPU3 > W-WPU1 > W-WPU2. As most macromolecular chains are very long with infinitesimally small cross profiles, they curl to form a ball-of-string morphology. Thus, some macromolecular chains can be observed as spherical particles via high-resolution TEM. In our research, all samples except for W-WPU2 exhibited this morphology. On one hand, the causes of the differences in the homogeneity, size and shape of the nanoparticles are of interest. On the other hand, the cause of the interesting shape shown by W-WPU2 is an attractive topic. The differences could be ascribed to the crimp degree and order degree of the polyurethane chains in the aqueous environment. In this study, the crimp degree and order degree of molecules should be strictly distinguished. We assume that the crimp degree (molecular self-assembly) of waterborne polyurethane chains is determined by the molecular weight (*M*_*n*_), resulting in different shapes that range from spheres to irregular elongated shapes, and the order degree of waterborne polyurethane chains is determined by the strength of the hydrogen bonds, determining the orientation and crystallinity of the chains. The GPC results reported above indicate that *M*_n_ (number-average molecular weight) occurred in the sequence I-WPU < IW-WPU < W-WPU3 < W-WPU1 < S-WPU < W-WPU2. The order of the *M*_n_ of the WPU samples was almost the reverse of the crimp degree and corresponding sphericity, suggesting that the nanoparticles change from spheres to irregular elongated shapes with increasing polyurethane molecular weight (*M*_n_). This result is consistent with the previous study^13^. The only exception was S-WPU, which may have arisen from the special structure and properties of snow. Regarding the order degree of the WPU samples, we consider S-WPU and W-WPU2 to have the highest order, while I-WPU takes the second place, and the other three samples (W-WPU3, IW-WPU, W-WPU1) are nearly identical, as shown in Fig. 3 and the XRD analysis. As shown in Fig. 4, the diffraction patterns of the WPU samples exhibited a dominant amorphous halo, with a weak shoulder appearing at the 2θ values of 42–43°, indicating a reduced degree of orientation structure that presumably arose from some minor level of hard segment organization^35^. Figure 4 shows that the peaks at 42–43° of S-WPU and W-WPU2 were more distinct than in other samples, suggesting a higher degree of orientation structure. In addition, a new weak peak corresponding to 2θ = ca. 29.5° was observed in the W-WPU2 curve in Fig. 4, which might be associated with the formation of a new orientation structure or crystalline phase in W-WPU2 according to previous work^36^. This sequence of the order degree of all WPU samples can also be observed in Fig. 3. W-WPU2 presented a perfect aggregation of tube shaped nanoparticles, which was also observed by Azaïs and Nassif *et al.*^24^ in studying the impact of water on the structure of bone apatite. They suggested that the tube-shaped nanoparticles of ~50 nm in size were irregular platelets, but we disagree. In aqueous solution, water can bring order to the water suspension of organics^14^,^16^. Thus, these nanoparticles of ~50 nm in size may be particles of some type of organics in water suspension. Our research suggests that the tube-shaped nanoparticles are formed by the crimping of polyurethane chains in aqueous solution. According to previous research, the order degree of the polymer is determined by the strength of the hydrogen bonds^17^,^37^. Meanwhile, FTIR analysis of the strength of the hydrogen bonds of the carbonyl region corresponded to the results of the order degree of all the WPU samples. Moreover, it seems that the homogenous dispersion and spherical particles may lead to a transparent emulsion, according to Fig. 3(a).

**Figure 5:**
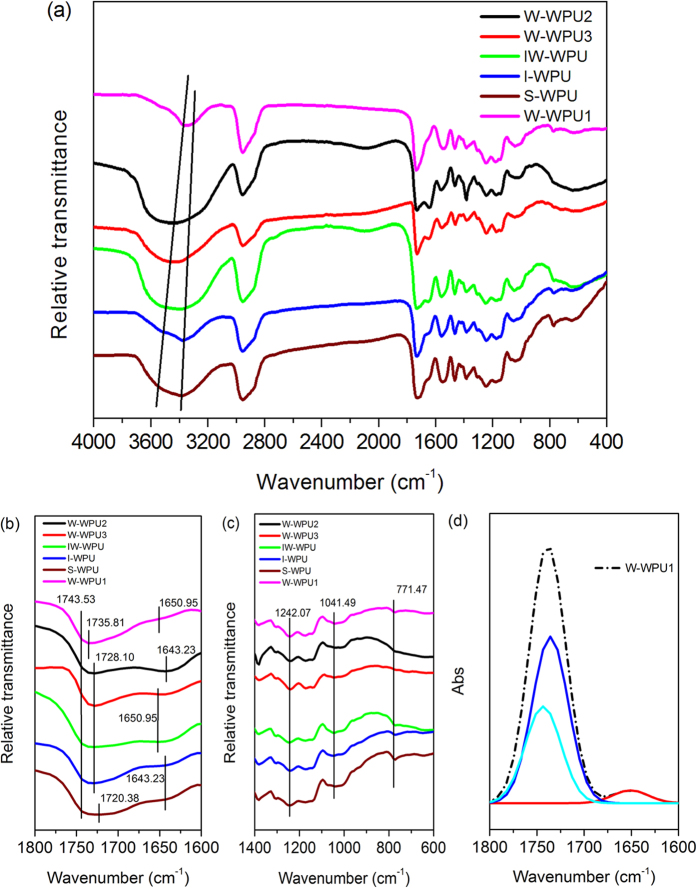
FTIR spectra of WPU samples synthesized from water in different form and temperatures (**a**) the whole region from 4000 cm^−1^ to 400 cm^−1^; (**b**) the main region for carbonyl group from 1800 cm^−1^ to 1600 cm^−1^; (**c**) the main region for ether group from 1400 cm^−1^ to 600 cm^−1^; (**d**) a split of carbonyl region between 1800 cm^−1^ and 1600 cm^−1^ of W-WPU1 for the absorbance model, which is converted from the transmittance model.

To further confirm the causes for the difference in WPU particles’ morphology, FTIR spectra were acquired, and the spectral range from 400 to 4000 cm^−1^ is displayed. As shown in Fig. 5(a), the FTIR spectra of the WPU samples were quite similar, exhibiting the typical curve of waterborne polyurethane^5^,^9^. Two transmittance bands from 3448.48-3332.76 cm^−1^ could be observed, and these were assigned to the urethane hydrogen bonded O-H and N-H vibration (amide I). The transmittance bands in the range of 1542.94–1558.37 cm^−1^ and at 771.47 cm^−1^ (shown in Fig. 5(c)) belong to the urea stretching C-N + bending N-H vibration (amide II) and aliphatic N-H plane bending vibration, respectively^38^. These representative FT-IR bands suggest the generation of polyurethane. Meanwhile, as the most important factor in band vibrational frequency shifts for polyurethane bulk structure, hydrogen bonds (H-bonds) have been found in the FTIR spectra of PU^39^,^40^. Due to the effects of the hydrogen bonds, the FTIR spectroscopy bands became wide, and the frequency of the functional group shifted substantially. According to Painter *et al.*^41^, the most hydrogen bond information can be obtained from the N-H and C=O stretching vibrations near 3200–3400 cm^−1^ and 1600–1700 cm^−1^, respectively. In the carbonyl region, there were three peaks attributed to C=O stretching vibrations from 1800-1600 cm^−1^ (the split peaks for the W-WPU1 sample are shown in Fig. 5(d)), which may be attributed to the transmittance of the carbonyl groups in the urethane, urea and ester groups of the WPU samples, as is clearly shown in Fig. 5(b). All the curves presented a weak band at 1743.53 cm^−1^, which was assigned to a free urethane carbonyl group^41^. The strong band at 1735.53 cm^−1^ for W-WPU1; at 1728.10 cm^−1^ for W-WPU2, W-WPU3, IW-WPU and I-WPU; and at 1720.38 cm^−1^ for S-WPU corresponding to a urethane hydrogen-bonded disordered carbonyl group and the medium peak at approximately 1645 cm^−1^ corresponding to a urea hydrogen-bonded disordered carbonyl group are distinctly visible in Fig. 5(b). Compared with W-WPU1, the decrease in frequency of the urethane hydrogen-bonded disordered carbonyl group of the other samples may have arisen from the stronger hydrogen bonds. In addition, the band at 1643.23 cm^−1^ for the urea hydrogen-bonded disordered carbonyl group of S-WPU, I-WPU, W-WPU2, with a lower frequency than the other three samples at 1650.95 cm^−1^, clearly indicates stronger hydrogen bonds in the urea carbonyl group. The results for the carbonyl region suggest that the WPU dispersions were successfully synthesized with various degrees of hydrogen bonds. The hydrogen bonds in S-WPU were the strongest, those in I-WPU and W-WPU2 took second place, those in IW-WPU and W-WPU3 were third and those in W-WPU1 were the weakest. This result is consistent with previous studies^42^,^43^. The different strengths of the hydrogen bonds in the WPU samples may have arisen from the different electron delocalization of the hydrogen donors in liquid water, ice and snow^37^. However, the effects of water in different forms with different structures and of hydrogen bonds on the polymer structure are extremely complex due to the polymorphism of crystalline ice and liquid water^44^. In addition, as is shown in Fig. 5(c), two sharp bands at 1242.07 cm^−1^ and 1041.49 cm^−1^ could be seen in all the WPU spectra, and these were ascribed to the symmetric and asymmetric stretching of the ether group in the soft segment. The impact of the hydrogen bonds on the morphology of WPU corresponds to the aforementioned analysis.

**Figure 6:**
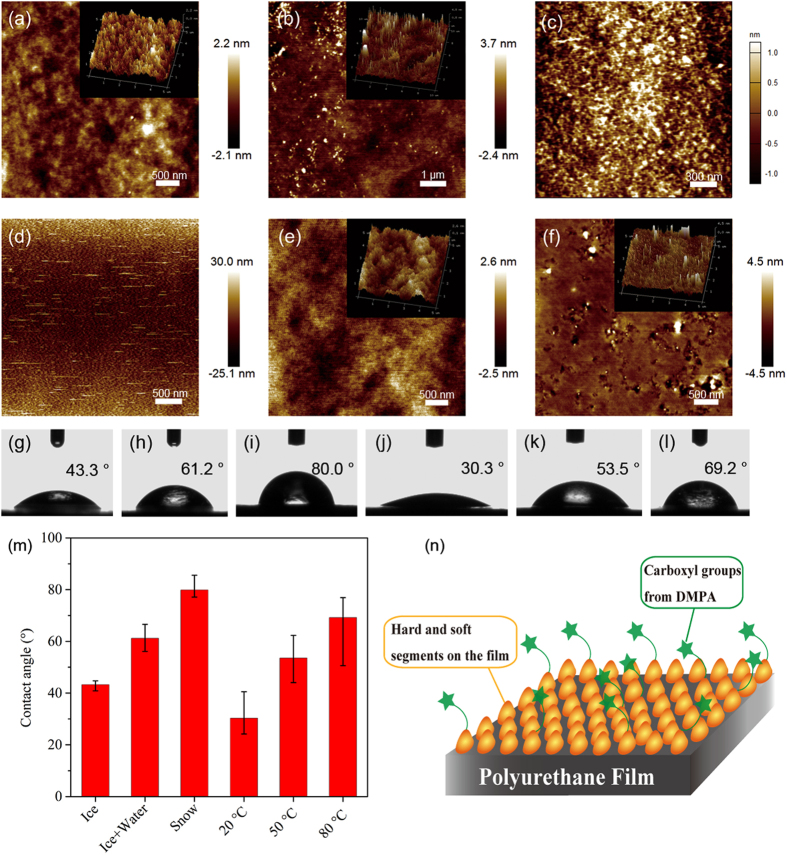
AFM images of the WPU films on a silicon substrate. (**a,b,e,f**) Height images shown for a 5 μm × 5 μm area for sample I-WPU, IW-WPU, W-WPU2 and W-WPU3, respectively; (**c**) Height image for a 2 μm × 2 μm area for sample S-WPU; (**d**) Height image for a 10 μm × 10 μm area for sample W-WPU1. The corresponding 3D images for the samples are inserted in the panels as the insets; (**g–m**) water contact angles and the tendency for the all samples; (**n**) cartoon for the polyurethane film surface.

AFM images of the WPU films are presented in Fig. 6. The bright regions, which are nanocylinders in 3D images and show nanometric scale hard globules in height images, were attributed to hard-segment nanocylinders dispersed within a soft segment, consisting of certain microphase separated structures according to previous research^45^,^46^. All images indicated a morphology consisting of generally spherical domains; these are most likely a combination of spherical domains and nanocylinders^46^, with spheres that differ from the spherical nanoparticles mentioned in the TEM experiment. As shown in Fig. 6, there were more spherical domains (the analogous spheres consisting of hard and soft segments in the cartoon of Fig. 6(n)) in (a) and (c) than in the rest, indicating that solid water can facilitate the formation of spherical domains and the dissolution of hard segments in soft segments. To further investigate the surface properties of polyurethane film, a water contact angle experiment was performed. As shown in Fig. 6(g–l), the water contact angles for all samples were below 90°, suggesting that the polyurethane films are hydrophilic. This result may have arisen from the existence of carboxyl groups (the hydrophilic groups in WPU) in the hard segments derived from DMPA, indicating that there are greater or fewer COO^−^ groups on the film surfaces, as depicted in the cartoon in Fig. 6(n). The S-WPU and W-WPU1 samples possessed the largest (80.0°) and smallest angles (30.3°), respectively, which were attributed to the smallest and largest amounts of COO^−^ groups, respectively. Thus, we assume that more hard segments gather on the surface than in other samples to supply more hydrophilic groups. The contact angles of all the samples varied dramatically (shown in Fig. 6(m)), indicating a significant tendency depending on the water form and temperature. Compared with the polyurethane obtained under normal conditions (W-WPU1), the contact angles increased when ice or snow was employed and when the water temperature was increased. Notably, snow can thaw to water more easily than ice, indicating different water addition speeds for I-WPU, IW-WPU and S-WPU synthesis. Among the three samples, the water addition speed was the highest for S-WPU (Snow in Fig. 6(m)) and lowest for I-WPU (Ice in Fig. 6(m)), corresponding to the tendency of water contact angles. This result may imply a correlation between the water addition speed and surface properties of the polyurethane films. The details will be further studied in future research.

**Figure 7:**
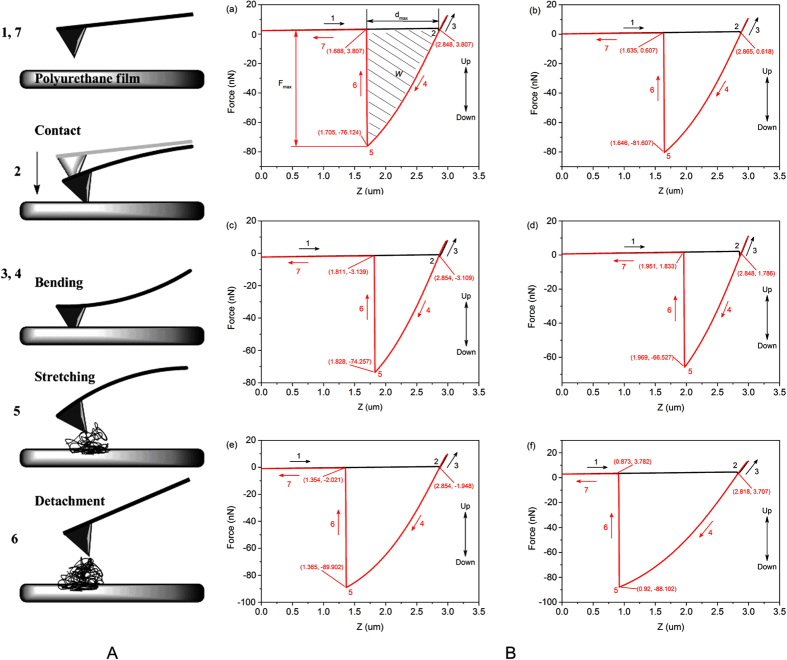
The diagram of nanomechanical properties of WPU by AFM. (**A**) Sketch of the seven stages reflecting the variation of WPU films during the force spectroscopy experiment. (**B**) (a–f) Experimental force-distance curves for a trace and retrace for samples I-WPU, IW-WPU, S-WPU, W-WPU1, W-WPU2, W-WPU3, respectively; the stages labeled on the curves corresponds to the sketch of seven stages. The meanings of *F*_max_, *d*_max_ and *W* are also identified in (a).

**Figure 8:**
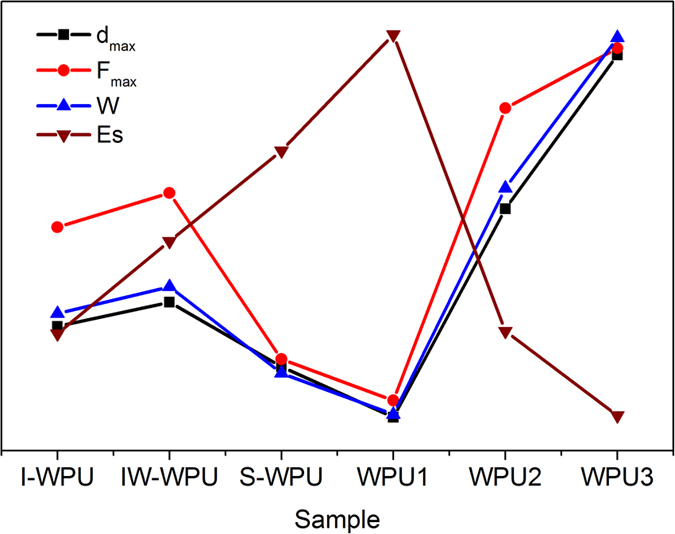


Force spectroscopy (FS) experiments are sensitive to nonlinear elastic properties at high strain. Meanwhile, the FS curves can provide valuable and significant information on local material mechanical properties, including adhesion, dissipation, Young’s modulus and deformation. Thus, FS curves have become essential in polymer research^47^ and are undoubtedly equally important in polyurethane research. The deformation of polyurethane films during adhesive debonding is reflected by the measurement of *d*_*max*_ values listed in Table 2, and the detection process is depicted in Fig. 7. The effect of water reacting with polyurethane prepolymer at different temperatures and in different forms is apparent. When the water was in liquid form, the *d*_*max*_ value of WPU3 was highest among WPU1, WPU2 and WPU3, indicating that greater deformation can be obtained by increasing the temperature of the liquid water reacting with the polyurethane prepolymer. According to Keddie *et al.*^28^, greater nanoscale deformation might be attributable to the fibrillation of the polyurethane films during the FS experiment. Thus, we suggest that liquid water at high temperature can facilitate fibrillation in polyurethane synthesis. Meanwhile, the *d*_max_ values of I-WPU and S-WPU were lower than for IW-WPU, indicating that the existence of liquid water can also facilitate fibrillation. The values of adhesion (*F*_max_) and adhesion dissipation (*W*) are presented in Table 2, and their variation tendency is depicted in Fig. 8. The highest *F*_max_ and *W* values occurred for WPU3, suggesting that high-temperature water contributes to high adhesion and viscoelasticity for polyurethane. Furthermore, the variation tendencies of *d*_max_, *F*_max_ and *W* were nearly identical (Fig. 8). Thus, the nanoscale deformation, adhesion and viscoelasticity for polyurethane films are proportional.

Notably, the Young’s modulus of WPU was complex compared to the other mechanical properties. According to previous research^29^,^47^, the main theories regarding elastic contact in FS curves are the Sneddon, Hertzian, JKR and DMT models, which apply to different materials with various performance and features. In this work, we selected the JKR model to calculate Young’s modulus for WPU because it is relatively accurate and gives reliable results^29^. Moreover, the JKR model is widely used to estimate the elastic modulus of soft materials that present spherical particles with large adhesion between the sample and tip^31^,^47^,^48^,^49^. As stated in the TEM results, five WPU samples showed spherical or suborbicular morphology, indicating that the JKR model is more suitable for comparing Young’s modulus among the polyurethane samples. In the JKR model, the effective Young’s modulus of the tip and polyurethane *E* is defined by




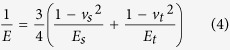




where *v*_*t*_, *E*_*t*_, *v*_*s*_ and *E*_*s*_ are Poisson’s ratio and Young’s modulus for the tip and WPU sample, respectively. For the polymer system in this work, we assumed that *E*_*t*_ ≫ *E*_*s*_ (the elastic modulus of the silicon tip is 160 GPa, versus 0.01–5 GPa for polymers generally) and *v*_s_ = 0.38 with a good accuracy^29^. Thus, we could approximate *E* as follows:




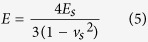




Meanwhile, the JKR model^50^ gives




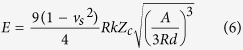







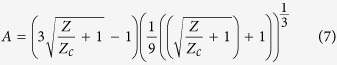




where *R* is the tip curvature radius, d is the tip penetration depth and A is the contact area. The only unknown is *Z*_*c*_, which can be acquired from the equation *F*_*A*_ = −*kZ*_*c*_. Based on the JKR theory, the adhesion force between the tip and sample is given by




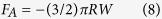




Thus, based on the adhesion of *W*, *Z*_*c*_ could be obtained. By combining equations (5) and (6), the *E*_*s*_ value could be obtained for all the WPU samples by




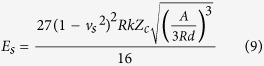




The *E*_s_ values of the WPU samples are shown in Table 2, which illustrates the stiffness of the polyurethane films. As depicted in Fig. 8, *E*_s_ was inversely proportional to *d*_max_, *F*_max_ and *W*, consistent with the characteristic that a higher Young’s modulus and a lower force correspond to a harder sample. This result suggests that the derived equation (9) is effective for obtaining and comparing Young’s modulus among the series of samples at nanoscale. It appears that liquid water at 20 °C results in the stiffest polyurethane film, indicating that more hard segments have gathered on the film surface to enhance the hardness of the film and corresponding to the water contact angle results. However, we must note that the calculations and values of the nanomechanical properties in this research can be used only to qualitatively compare the variation within a series of samples.

## Conclusions

Water in different forms and at different temperatures can cause various nanoparticle morphologies for waterborne polyurethane dispersions. We suggest that liquid water may disturb the formation of polyurethane chains into spherical particles, while solid water can facilitate the formation of spherical particles. Our results imply that the crimp degree is decided by the molecular weight of the polyurethane, which is ascribed to the isocyanate-water reaction. The 50 °C water leads to the highest molecular weight and narrowest distribution for polyurethane chains, and when the water temperature decreases, the molecular weight also decreases. Moreover, the strength of the hydrogen bonds is responsible for the order degree of the polyurethane nanoparticles. We suggest that the hydrogen bonds in W-WPU2 and S-WPU are the strongest, resulting in the attractively perfect and homogenous tube and sphere shapes, respectively. Furthermore, solid water and high-temperature water may reduce the gathering of hard segments on the polyurethane film surface, thus reducing hydrophilic groups and leading to a softer surface than the standard sample (W-WPU1). In summary, this work reveals an unknown role for water in different forms and at different temperatures that can affect the nanoparticle shape and surface properties of waterborne polyurethane.

## Additional Information

**How to cite this article**: Zhou, X. *et al.* Various nanoparticle morphologies and surface properties of waterborne polyurethane controlled by water. *Sci. Rep.* **6**, 34574; doi: 10.1038/srep34574 (2016).

## Figures and Tables

**Table 1:** Average molecular masses and polydispersity index of the polyurethanes.

Molecular weight	S-WPU	I-WPU	IW-WPU	W-WPU1	W-WPU2	W-WPU3
*M*_n_ (g/mol)	12722	9246	9482	12136	15657	11976
*M*_w_ (g/mol)	35061	29791	26004	35498	35288	31875
*M*_w_/*M*_n_	2.756	3.222	2.742	2.925	2.254	2.662

**Table 2:** Nanoscale mechanical properties of the WPU films by force spectroscopy experiments.

Sample	*d*_max_ (μm)	*F*_max_ (nN)	*W* (10^−15^ J)	*E*_*s*_ (10^−3^ MPa)
I-WPU	1.16	79.931	92.720	207.697
IW-WPU	1.23	82.214	101.123	297.799
S-WPU	1.043	71.118	74.176	386.045
WPU1	0.897	68.360	61.320	499.108
WPU2	1.5	87.881	131.822	210.302
WPU3	1.945	91.884	178.714	127.970
