# Author Correction: Various nanoparticle morphologies and surface properties of waterborne polyurethane controlled by water

**DOI:** 10.1038/s41598-020-63069-6

**Published:** 2020-04-10

**Authors:** Xing Zhou, Changqing Fang, Wanqing Lei, Jie Du, Tingyi Huang, Yan Li, Youliang Cheng

**Affiliations:** 1 School of Mechanical and Precision Instrument Engineering, Xi’an University of Technology, Xi’an, 710048 P. R. China; 2 Faculty of Printing, Packaging Engineering and Digital Media Technology, Xi’an University of Technology, Xi’an, 710048 P. R. China; 3 College of Art and Design, Xi’an University of Technology, Xi’an, 710048 P. R. China; 4 School of Civil Engineering and Architecture, Xi’an University of Technology, Xi’an, 710048 P. R. China

Correction to: *Scientific Reports* 10.1038/srep34574, published online 30 September 2016

This Article contains an error.

In the Results and Discussion section, the Authors discussed work described in Ref. 24 in a way that suggest that aggregation of tube-shaped nanoparticles was caused by the water-suspended organic molecules. However, system described in Ref. 24 was devoid of organic molecules. To clarify this in the text,

“W-WPU2 presented a perfect aggregation of tube shaped nanoparticles, which was also observed by Azaïs and Nassif et al. [24] in studying the impact of water on the structure of bone apatite. They suggested that the tube-shaped nanoparticles of ~50 nm in size were irregular platelets, but we disagree. In aqueous solution, water can bring order to the water suspension of organics [14, 16].”

should read:

“W-WPU2 presented a perfect aggregation of tube shaped nanoparticles. Previous studies demonstrated that water can bring order to the water suspension of organics [14,16] and inorganics [24] in aqueous solution. Thus, these nanoparticles of ~50 nm in size may be some type of organic particles in the water suspension.”

